# Primary Signet Ring Cell Carcinoma of the Lung with Cerebellar Metastasis Showing Full Response to Cisplatin and Docetaxel Therapy

**DOI:** 10.1155/2014/968723

**Published:** 2014-01-09

**Authors:** Onur Kocas, Fatih Selcukbiricik, Ahmet Bilici, Metin Kanıtez, Serdar Yildiz, Suna Avci, Canan Tanik

**Affiliations:** ^1^Division of Internal Medicine, Sisli Education and Research Hospital, 34200 Istanbul, Turkey; ^2^Division of Medical Oncology, Department of Internal Medicine, Sisli Education and Research Hospital, 34200 Istanbul, Turkey; ^3^Division of Pathology, Sisli Education and Research Hospital, 34200 Istanbul, Turkey

## Abstract

*Introduction*. Primary signet ring cell carcinoma (SRCC) of the lung is a very rare disease. We describe a new case of primary SRCC of the lung with cerebellar metastasis, which responded well to the therapeutic approach with cisplatin and docetaxel. *Case Report*. A 41-year-old female patient (nonsmoker) was consulted to our oncology outpatient clinic after cerebellar metastasectomy. The histopathological diagnosis was SRCC metastasis. The primary tumor was unknown. The PET-CT imaging showed a hypermetabolic mass in the right middle lobe of the lung and hypermetabolic mediastinal lymph node stations. Oesophagogastroduodenoscopy and colonoscopy showed no evidence of gastrointestinal system tumor. The clinical diagnosis of primary SRCC of the lung was made and the administration of six rounds of cisplatin and docetaxel treatment was planned. After the chemotherapy the PET-CT scan to evaluate the therapy response showed full metabolic regression of the primary tumor and the mediastinal lymph nodes. There was no evidence of new metastasis. *Conclusion*. Primary SRCC of the lung is a very rare disease with poor prognosis. There are not many cases in literature and no standardized chemotherapy protocols. Cisplatin and docetaxel may be a good treatment option.

## 1. Introduction

Primary SRCC of the lung is a very rare disease. First described by Kish et al. in 1989, it is reported that incidence of primary SRCC of the lung varies from 0.14% to 1.9% of all lung cancers [1]. The largest series was taken by Tsuta et al. in which 39 of 2640 surgically resected primary lung carcinomas showed SRCC components. Mean age of the patients was 54.6 years, male to female ratio was 1.16 : 1.00, and 26 patients (66.7%) were smokers. The size of the SRCC component of the tumor positively correlated with the aggressiveness of the tumor and poor outcome. The 5-year survival was 28% [2].

## 2. Case 

**Figure 1:**
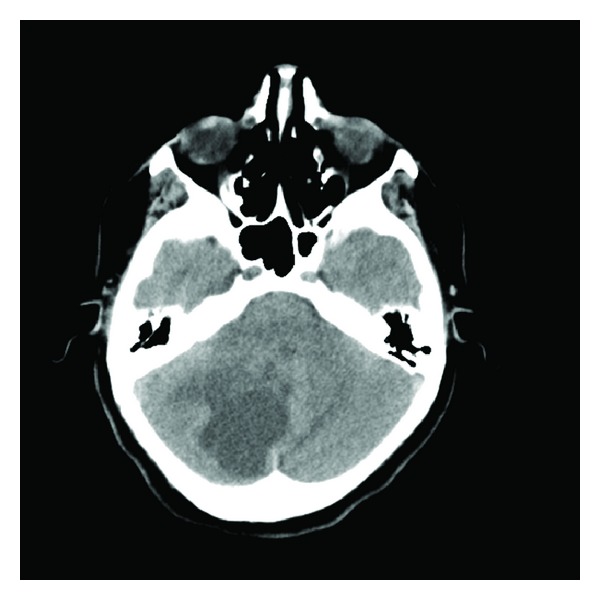
The preop CT scan showed a right cerebellar hypodens lesion.

A 41-years-old female patient (nonsmoker) was consulted to our oncology outpatient clinic by the neurosurgery clinic after a cerebellar metastasectomy. The preop CT scan showed a right cerebellar hypodense lesion with the dimension of 5 × 5 cm (Figure 1).

**Figure 2:**
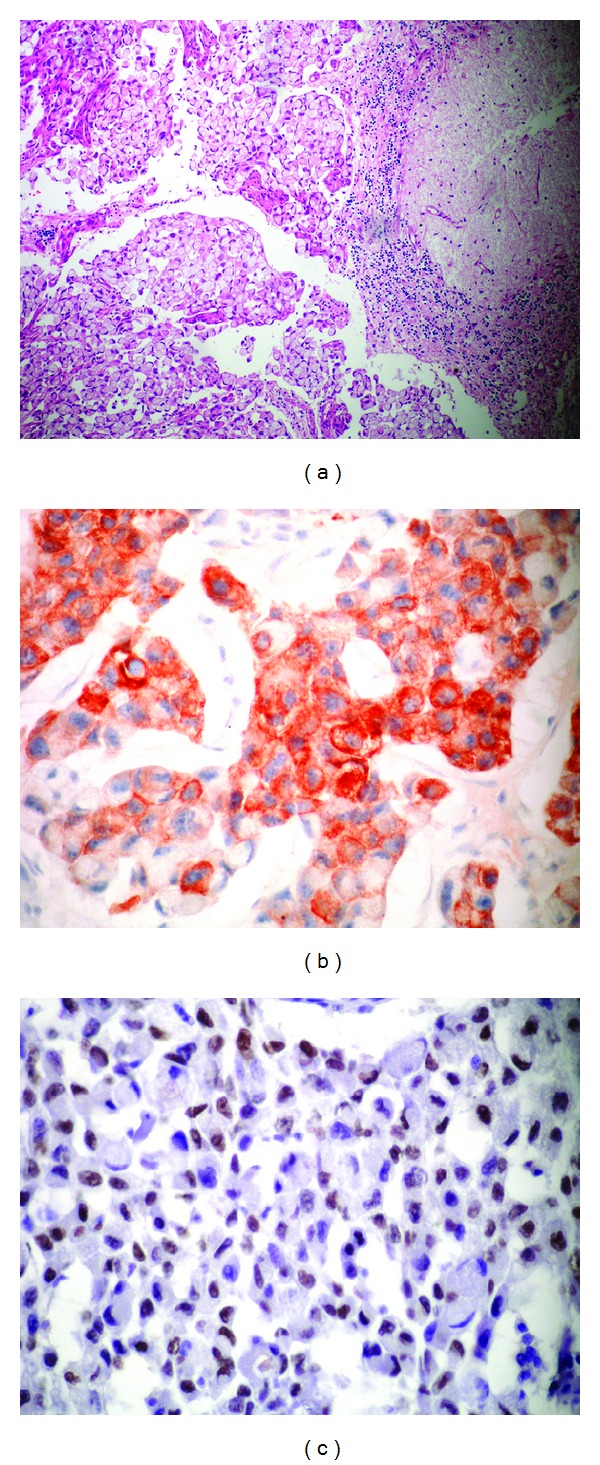
(a) Malign epithelial infiltration in the cerebellum with signet-ring cell morphology. HE x100. (b): Cell groups in the tumor show positive staining with cytoplasmic CK 7. CK7 x200. (c) Positive nuclear staining with TTF-1 in the signet-ring tumor cells. TTF-1 x200.

The histopathological examination of the metastasectomy material showed SRCC metastasis with positive immunostaining for CEA, CK 7, and TTF-1. The immunostaining with CK 20, ER, COX2, CK 14, CDX2, and MUC2 was negative. The histopathological morphology was shown Figures 2(a), 2(b), and 2(c).

In order to find the primary tumor a PET-CT scan, oesophagogastroduodenoscopy and colonoscopy was planned. The endoscopic evaluation of the gastrointestinal system showed no evidence of tumor. The PET-CT scan showed a 26 × 23 mm sized hypermetabolic lesion (SUVmax: 12.1) in the right middle lobe of the lung, multiple 10 mm sized hypermetabolic lymph nodes (SUVmax: 5.7) at station 10R, and a 21 × 19 mm sized hypermetabolic (SUVmax: 12.4) lymph node at station 11R in the mediastinum. The clinical diagnosis was primary SRCC of the lung. The patient received cranial radiotherapy. After the radiotherapy we decided to administrate the patient six rounds of cisplatin and docetaxel regimen. The PET-CT scan to reevaluate the therapy after the 3 rounds of the chemotherapy showed decreased FDG uptake (SUVmax: 5.1) and a decrease in the size of the primary lesion, which we interpreted as partial regression. The hilar metastatic lymph nodes showed nearly full regression and could barely be visualized in the PET-CT images. We decided to complete the cisplatin and docetaxel protocol. The PET-CT scan after the completion of the chemotherapy showed a decrease in size (19 × 25 mm) and metabolic regression (SUVmax: 2.6) of the primary tumor in the right middle lobe of the lung, there was no hypermetabolic pathological lymphadenopathies in the mediastinum. In the left axilla, a 7 × 14 mm sized nodular lesion with slightly increased FDG uptake (SUVmax: 1.3) was detected. There was no pathological FDG uptake in the rest of the body.

## 3. Discussion

Primary SRCC of the lung is a very rare disease. The largest series was taken by Tsuta et al. in which 39 of 2640 cases surgically resected primary lung carcinomas showed SRCC components. Mean age of the patients was 54.6 years, male-to-female ratio was 1.16 : 1.00, and twenty six patients (66.7%) were smokers. The size of the SRCC component positively correlated with the aggressivenes of the tumor and poor outcome. The 5-year survival was 28% [2].

Because of the rareness of the disease, it is important to distinguish the primary SRCC of the lung from metastatic SRCC's from other sites of the body like stomach, colon, breast, urinary tract which are more common.

Immunohistochemical studies and molecular diagnostics should help in making the differential diagnosis. The studies of Merchant et al. with 32 SRCC's from various organs (17 lung, 5 breast, and 5 stomach and 5 colon) showed that 82.4% of pulmonary SRCC's were TTF-1 positive and the cytokeratine profile CK 7 +/CK20 - was identified in 94.1% of the pulmonary SRCC cases. Villin was in 29.4% of the cases positive [3]. Positivity for both TTF-1 and Napsin-A is a strong indicator for pulmonary origin [4, 5].

Hayashi et al. reported 5 cases of primary lung SRCC. 80% of the cases were immunoreactive for lactoferrin, 100% showed K-Ras mutations, 100% were positive for MUC 1 and 100% were negative for MUC 2. MUC 1, is most commonly seen in SRCC and the solid adenocarcinoma of the lung than in SRCC of other organ sites [6].

Although uncommonly, TTF-1 can also be expressed in carcinomas originating from other primary sites (e.g., colon) [7]. On the other side negativity for TTF-1 does not exclude pulmonary SRCC if the tumor is CK 7, positive. In this case metastatic tumor from other body sites need to be excluded clinically [8]. In our case the tumor was positive for CEA, CK 7 and TTF-1 and negative for CK 20, ER, COX2, CK 14, CDX2, and MUC2. It should also be noted that in 70% of pulmonary adenocarcinomas ALK gene rearrangement can be identified, when the SRCC component is >10% [9]. Shaw et al. demonstrated that ALK thyroxine kinase inhibitor (crizotinib) therapy has positive impact on improved survival [10].

A comprehensive, retrospective population-based analysis of the primary SRCC of the lung with the adenocarcinoma of the lung by Ou et al. showed that the patients with primary SRCC are significantly younger than patients with adenocarcinoma, with a significantly higher proportion of poorly differentiated tumor and stage IV disease and no difference in the distribution of gender and ethnicity. Never smokers comprised a higher proportion of patients with SRCC (30.8%) compared to patients with adenocarcinoma (11.0%). Never smokers with SRCC were younger and had an improved overall survival (median age: 55 years, median overall survival: 8 months) than smokers with SRCC (median age: 59 years, median overall survival: 4.5 months). Patients with SRCC had decreased overall survival compared to adenocarcinoma patients [11]. Our patient is a 41-year-old, female, never smoker. Seven months after diagnosis and six rounds of cisplatin and docetaxel therapy, she is well and alive with a satisfactory response to chemotherapy.
